# Development, Validation, and Application of the Paya Hamsan Technologies Underivatized Newborn Screening Assay (PHUNSA) for Inborn Metabolic Disorders in Dried Blood Spot Samples from Iranian Infants

**DOI:** 10.3390/ijns11010004

**Published:** 2025-01-08

**Authors:** Azam Khodadadi, Saber Nanbedeh, Mahsa Joodaki, Bradford L. Therrell, Kambiz Gilany

**Affiliations:** 1 Molecular Engineering & Sciences Institute, Arak University, Arak 3848177584, Iran; n.khodadadi65@gmail.com (A.K.); snanbedeh@gmail.com (S.N.); joodaki.mahsa@gmail.com (M.J.); 2 USA National Newborn Screening and Global Resource Center, Austin, TX 78759, USA; therrell@uthscsa.edu; 3 Department of Pediatrics, University of Texas Health Science Center at San Antonio, San Antonio, TX 78229, USA; 4 Integrative Oncology Department, Breast Cancer Research Center, Motamed Cancer Institute, ACECR, Tehran 1517964311, Iran; 5 Reproductive Immunology Research Center, Avicenna Research Institute, ACECR, Tehran 1983969412, Iran

**Keywords:** inborn metabolic disorders, newborn screening, tandem mass spectrometry, dried blood spot, non-derivatized assay

## Abstract

Screening for inborn metabolic disorders (IMDs) in newborns is an important way to prevent serious metabolic and developmental difficulties that can result in lasting disabilities or even death. Electrospray ionization tandem mass spectrometry (MS/MS) provides an efficacious newborn blood spot screening (NBS) mechanism for analyzing dried blood spot specimens (DBSs) for biochemical markers for these conditions. Where possible, the elimination of derivatization in specimen preparation can simplify and streamline analysis. The Paya Hamsan Technologies Underivatized Newborn Screening Assay (PHUNSA) is an underivatized MS/MS test kit for IMD NBS. Validation of the accuracy, precision, linearity, and stability was based on the ISO 15189 standard and the CLSI NBS04 guideline. The PHUNSA kit demonstrated suitable performance along with acceptable recovery rates and negligible bias for many IMD analytes. Assay sensitivity was demonstrated through acceptable limits of detection (LOD) and lower limits of quantification (LLOQ). Specimen preparation times were decreased, the coefficients of variation were consistently below 10%, and accuracy and stability were demonstrated under various testing conditions, including prolonged storage and transportation. The PHUNSA kit provides a simplified, efficient, and reliable approach to IMD NBS with the potential to enhance NBS in Iran and other locations by providing a scalable, cost-effective, and streamlined option for early IMD detection and management.

## 1. Introduction

Untreated inborn metabolic disorders (IMDs) can cause neurological damage, physical disability, and even death due to the abnormal levels of metabolites involved in important physiological processes [1]. A combination of genetic factors and high consanguinity increases the risk for IMDs in Iran and underscores the need to develop effective and efficient screening procedures. Newborn blood spot screening (NBS) allows the prevention of the serious metabolic and developmental problems that can result from IMDs and that may cause permanent impairment or even death [2]. Through early detection and disease management, newborns and their families can experience improved quality of life and correspondingly reduce the strain on national healthcare resources.

Alongside the growth of NBS, the emergence of modern technologies like electrospray ionization tandem mass spectrometry (MS/MS), with its enhanced diagnostic capacity, has significantly contributed to expanded NBS for IMDs, thus improving newborn healthcare worldwide [3]. Using MS/MS technology, it is possible to simultaneously quantify a wide range of metabolites using only a single dried blood spot (DBS). Consequently, screening and diagnostic processes are accelerated, and disease management can begin earlier. Specimen preparation for MS/MS can be streamlined by using non-derivatized specimens, which eliminates a sample preparation step in the analysis protocol used in conventional derivatization MS/MS methodologies. This improved laboratory efficiency reduces the risk of errors during sample handling and assay preparation, which increases assay reliability [2].

This report describes the development and validation of the Paya Hamsan Technologies Underivatized Newborn Screening Assay (PHUNSA) for IMDs in DBS specimens (Paya Hamsan Technologies Co., Arak, Iran). Beyond improving Iran’s NBS system, the PHUNSA kit provides a model IMD NBS process for use in other locations with similar genomic and socioeconomic profiles. The incorporation of this assay into NBS systems is a scalable, efficient, and cost-effective way to solve a pressing public health problem [4]. The correct application of this screening test has the potential to impact metabolic disease management globally. Better understanding the prevalence and types of metabolic disorders in Iranian infants will also inform metabolic disease research, advance early treatments, and improve disease outcomes [5].

## 2. Materials and Methods

### 2.1. Study Design

The objective of this study was to utilize the newly developed PHUNSA kit (PH NBS complete kit, order no. PH 2001) to analyze DBSs from Iranian newborns for the presence of indicators of various IMDs. The assay’s capacity to detect IMD-related biomarkers was validated using specimens prepared without the use of derivatization techniques.

### 2.2. Ethical Considerations

The study was approved by the Research Ethics Committees of Avicenna Research Institute under registration code IR.ACECR.AVICENNA.REC.1403.006.

### 2.3. Chemicals and Reagents

A non-derivatized reagent kit specifically intended for the analysis of amino acids and acylcarnitines by liquid chromatography tandem mass spectrometry (LC-MS/MS) was used in the study. By eliminating the derivatization step, the analytical procedure is simplified and the potential for specimen handling errors is minimized. Essential components, such as mobile phase, calibration standards, and quality control samples, were included in the kit for direct DBS analysis on our analytical platform.

### 2.4. Analytical Equipment Details

Quantitative analysis was performed using an AB Sciex 3200 Mass Spectrometer (SCIEX, Concord, ON, Canada) equipped with an electrospray ionization (ESI) source. Data acquisition and analysis were managed using SCIEX Analyst Software version 1.6.3, which gave the robust data processing capabilities required for accurate quantification and reliability.

All laboratory equipment was routinely calibrated and serviced in compliance with the manufacturer’s specifications and industry standards to guarantee consistent performance throughout the study.

### 2.5. Kit Performance Comparison and Quality Assurance

A side-by-side comparison was conducted between the PHUNSA kit and the CE-certified ChromSystems kit (Munich, Germany) used nationwide in Iran. The comparison followed the CLSI NBS04 guideline for newborn screening by tandem mass spectrometry [6] and the ISO 15189 standard for medical laboratories [7]. To meet the Ministry of Health and Medical Education’s (MOHME) strict validation standards, assays were performed by specialized laboratories using the MS/MS system, in line with the ministry’s directive (HD-IMD-00-MN-SD-006-001).

### 2.6. Sample Collection and Preparation

Newborn blood was obtained by heel prick and absorbed onto pre-labeled Whatman 903 filter paper cards in compliance with the CLSI NBS01 standard, Dried Blood Spot Specimen Collection for Newborn Screening [8]. Each card was given a unique laboratory identification number for traceability.

### 2.7. Analytical Methods

A 3.2 mm disc was punched from each collection card and inserted into a 96-well flat-bottom plate. Then, 100 µL internal standard (reconstituted with extraction buffer (methanol/water)) was added to each sample. The analytes were extracted by shaking for 30 min at 700 rpm, and the supernatant was transferred to a 96-well conical-bottom plate for MS/MS analysis. Table S1 provides a list of the analytes, their associated quantitative parameters, and the details for each transition. Analyte concentrations were determined using standards in similar matrices. To ensure accuracy and reliability, quality control (QC) samples were analyzed with each batch.

### 2.8. Accuracy and Precision Measurements

Accuracy was assessed at individual and multiple testing locations following the recommendations in CLSI EP05-A3 [9]. Testing was performed over 20 days twice daily using two levels of DBS controls (Supplied by Paya Hamsan Technologies) to assess and ensure kit precision.

Recovery analyses were conducted in accordance with CLSI NBS04. Intra-assay variability was assessed by performing each test in duplicate across five independent working sites over several days. To simulate routine screening conditions, two concentration levels (Level I and Level II) of DBS were used as control samples. Acceptable recovery rates, defined as 40–140%, were established based on control sample guidelines.

The intra-lab precision and reproducibility were evaluated by obtaining multiple measurements of control samples over a period of 20 days. Measurements were taken to evaluate consistency, variances between runs, variations within a single day, and variations across days. Data analysis was conducted using a two-way nested ANOVA process. Over the course of five days, various operators from different laboratories used two separate LC-MS/MS machines to assess the inter-laboratory precision across more than one testing site. A two-way nested ANOVA was used to assess repeatability and consistency between different instruments, using two different concentration levels of controls from Paya Hamsan Technologies.

To demonstrate the diagnostic efficacy of the non-derivatized test versus traditional derivatized procedures, analyses using the PHUNSA kit were compared to the MassChrom kit to validate its reliability, accuracy, and effectiveness. Both kits were used to examine control samples and assess any measurement variations while maintaining kit consistency. The primary focus was on each assay’s ability to precisely detect and measure pertinent metabolites.

The limit of detection (LOD) and the lower limit of quantification (LLOQ) were determined by dilution of the prepared dried blood samples with an extraction buffer/internal standard at various ratios (1:10, 1:20, 1:50, 1:100, 1:200, 1:500, and 1:1000) and evaluated using the AB SCIEX 3200 apparatus on five separate occasions. The %CV was computed for each analyte. The LOD was determined as the concentration level at which the %CV reached 25%. The LLOQ was determined by multiplying the LOD by a factor of three.

The degree of linearity, the capacity to endure changes in concentration levels, and the ability to analyze stored specimens (including freeze–thawed and specimens stored long-term) were also determined along with testing reliability and accuracy and precision across different laboratories. Multiple repetitions of the tests were conducted across different variables (times, instruments, and operators) to evaluate the consistency within each study and to ensure accurate recovery rates. Statistical analyses were carried out using Microsoft Office Excel 2019 with a *p*-value of less than 0.05 defining statistical significance.

## 3. Results

### 3.1. Accuracy and Precision

The accuracy assessments revealed minimal bias across all the analytes. The recovery data in Table S2 demonstrate the efficacy of the PHUNSA kit. With one exception, all the recoveries equaled or exceeded 75% and were well within the 40–140% noted as being suitable in CLSI NBS04. Several recoveries exceeded 100%, indicating increased sensitivity for these indicators, further confirming the clinical viability of the underivatized assay.

Exceptional reproducibility was observed both within and between assays, with coefficients of variation (%CV) consistently below 10%. Intra-lab precision was consistent throughout the 20-day assessment period, demonstrating a high degree of reliability (Table S3).

Multi-site precision was evaluated by analyzing DBS controls at two different concentrations (supplied by Paya Hamsan Technologies) (see Table S4). Two different instruments (each one an operator) were used in different laboratories over a period of 5 days with five replications per day. The data provide a comprehensive overview of the repeatability and variability metrics for the relevant analytes. The low coefficients of variation imply high precision. The inter-day and intra-instrument data indicate analytical stability and consistency over time.

### 3.2. Method Comparison Using Control Samples

Comparative data between the PHUNSA kit and the MassChrom kit are shown in Tables S5 and S6. Mean values, %CV, and percentage deviation from the target concentrations were determined for a comprehensive range of amino acids and acylcarnitines. The PHUNSA kit values were found to be in good agreement with the MassChrom kit, indicating satisfactory comparative performance.

The precision of the PHUNSA kit versus the MassChrom kit was evaluated by analyzing variations in the desired concentrations for a wide range of analytes. With the ChromeSystems kit values as a reference, the percentage deviations of the mean values were compared (see Table 1). Most of the analytes displayed deviations within the acceptable range, reinforcing the assays’ capability to deliver precise and consistent results.

### 3.3. Comparative Analysis Using Real Samples with Non-Derivatized Preparation Method

Forty real specimens from Iranian newborns were evaluated using both the PHUNSA kit and the MassChrom kit. The percentage deviation was computed for each analyte, with the Chrome Systems kit serving as the reference standard. A wide range of analytes was analyzed including both normal specimens and specimens from newborns suspected of having a metabolic disorder, and the data are displayed in Table S7. As shown in the percent deviation column, all the values were within the range of +30% to −30%, indicating statistically significant results.

### 3.4. Comparison of the Identification of Clinical IMDs

Immediately following validation of the PHUNSA kit, a comparison study was carried out on nine clinical specimens from patients suspected of having a metabolic condition. A number of disorders were identified, including TYR (Tyrosinemia), MET (Hypermethioninemia), PKU (Phenylketonuria), MSUD (Maple syrup urine disease), NKH (Nonketotic hyperglycinemia), CPT1A (Carnitine palmitoyltransferase 1 deficiency), PA (Propionic acidemia), and MCAD (Medium-chain acyl-CoA dehydrogenase deficiency), and MMA (Methylmalonic acidemia). Both kits performed equally well in detecting disease-specific markers in the true clinical specimens. The analytical results from both kits are shown in Table 2.

### 3.5. Limit of Detection (LOD) and Lower Limit of Quantification (LLOQ)

To assess the PHUNSA kit’s sensitivity, the LOD and LLOQ were determined for each sample. The LOD is the smallest concentration that the method can identify with a certain level of trust, and the LLOQ is the lowest concentration that can be quantified with a specific level of precision and accuracy (see Table S8).

### 3.6. Linearity and Method Robustness

The PHUNSA kit was evaluated for linearity and robustness. Typically, the correlation values exceed 0.99, indicating a strong ability to accurately measure changing analyte concentrations. The repeatability results consistently demonstrated a coefficient of variation (%CV) of less than 10% for all the investigated analytes, indicating technical reliability (Table S9).

### 3.7. Blank Test Analysis

Blank filter papers were prepared like patient samples and underwent 12 rounds of processing. The blank filter paper samples had to have measurements below the LLOQ to ensure assay accuracy and reliability. The blank samples were analyzed 12 times using the PHUNSA kit and the AB SCIEX 3200. The analytical results using blank filter paper are shown in Table S10. The mean values of the blank filter paper collection cards were consistently below the LLOQ.

### 3.8. Carryover

The influence of specimens with high analyte concentrations on the analysis of a following specimen (carryover) was investigated by testing the ‘memory’ effect. Carryover is mostly caused by the autosampler injection port contamination or contamination of the tubing that leads to the electrospray ionization source. To measure the carryover effect, samples of blank filter paper were analyzed immediately following the containing of a very high concentration of the analyte, e.g., a high-concentration control. Analysis was completed five times with the PHUNSA kit (Table S10).

### 3.9. Stability

The specimen stability was evaluated under various conditions to ensure consistent behavior throughout analysis. The specimens stored at −18 °C remained unchanged for 12 months. After simulated transit conditions, no significant degradation was observed. When kept at room temperature for up to 24 h, the specimens-maintained reliability and uniformity. Stability testing across different conditions confirmed consistent performance throughout analysis, including handling and transport. The stability data, including accelerated testing results, are available in the Supplementary Materials (Tables S11–S14).

## 4. Discussion

We demonstrated the satisfactory performance of the PHUNSA kit for the first time through comprehensive validation using internationally recognized protocols, meeting rigorous criteria for accuracy, precision, linearity, and stability. Low CVs during intra-assay, inter-assay, and inter-laboratory studies have demonstrated that the assay consistently exhibits high recovery rates with little bias for amino acids and acylcarnitines. The assay repeatability and accuracy data confirm its suitability for routine clinical usage, especially in NBS programs where precise and rapid diagnoses are crucial.

The PHUNSA kit simplifies NBS laboratory workflows by eliminating the time-consuming derivatization step, thus improving operational efficiency and lowering costs. This is particularly appealing and beneficial in high-throughput screening environments where speed and accuracy are crucial.

Our study findings provide strong evidence of the accuracy and precision of the PHUNSA kit. However, it goes beyond merely confirming the technical performance of the kit, offering evidence of its significance, potential for growth, and cost-effectiveness in real-world settings. Successful implementation of the PHUNSA kit in Iran may have implications for public health globally, if it is utilized in other similar settings [10]. Further research should also explore the long-term impacts on newborns identified by NBS with the PHUNSA kit, including provision of medical care and overall health outcomes.

## Figures and Tables

**Table 1:** Comparative deviation analysis of PHUNSA and MassChrom kits (n = 40).

Amino Acids	Level I			Level II			Carnitines	Level I			Level II		
	MassChrom	PHUNSA	%Dev	MassChrom	PHUNSA	%Dev		MassChrom	PHUNSA	%Dev	MassChrom	PHUNSA	%Dev
Alanine	609.804	580.558	−4.796	660.857	655.324	−0.837	(C0)	42.582	49.610	16.505	80.407	97.826	21.663
Arginine	58.694	59.410	1.220	174.684	180.980	3.604	(C2)	18.581	19.820	6.669	49.406	53.883	9.062
Aspartic acid	197.836	206.891	4.577	379.413	422.585	11.379	(C3)	3.844	4.318	12.333	10.573	12.123	14.655
Citrulline	65.331	75.484	15.542	216.204	254.518	17.721	(C4)	0.822	0.977	18.865	3.675	4.493	22.256
Glutamic acid	828.699	836.826	0.981	872.958	918.972	5.271	(C5)	0.470	0.493	4.902	1.974	2.098	6.284
Glycine	454.322	497.350	9.471	608.857	693.996	13.983	(C5DC)	0.592	0.657	10.981	2.034	1.700	−16.377
Leucine	396.118	441.772	11.525	541.907	626.707	15.648	(C6)	0.422	0.469	10.972	1.847	2.134	15.566
Methionine	88.850	93.516	5.251	214.392	237.415	10.739	(C8)	0.478	0.525	9.978	1.895	2.178	14.951
Ornithine	514.958	531.038	3.123	699.585	726.364	3.828	(C10)	0.444	0.474	6.578	1.800	2.026	12.560
Phenylalanine	238.650	241.500	1.194	489.750	517.221	5.609	(C12)	0.386	0.413	7.146	1.754	1.954	11.423
Proline	410.694	452.146	10.093	729.149	823.946	13.001	(C14)	0.409	0.446	9.168	1.710	1.960	14.619
Tyrosine	215.284	208.184	−3.298	469.148	474.932	1.233	(C16)	3.806	4.250	11.665	9.955	11.664	17.173
Valine	309.810	356.704	15.136	453.495	543.986	19.954	(C18)	2.720	2.365	−13.022	8.903	8.040	−9.693

**Table 2:** Comparative results from the PHUNSA and MassChrom kits for patients with markedly out-of-range values.

Patient ID	Metabolite	Possible Clinical Condition	Reference Interval	Alert Range	Results from MassChrom	Results from PHUNSA	Comments
2923	Tyrosine	TYR	<292.74	>336.58	467.00	535.63	Out of range
4428	Tyrosine	TYR	<292.74	>336.58	382.32	423.91	Out of range
4063	Methionine	MET	6.97–24.8	> 28.5, <6.34	193.10	230.33	Out of range
4060	C3	PA/MMA	0.37–4.30	> 5.0, <0.31	10.41	15.31	Out of range
4047	Multiple (C0, C16, C18, C18:1)	CPT1A	7.14–43.34 0.41–6.9 0.19–1.71 0.33–2.33	>48.0, <5.6 >7.13, <0.33 >1.89, <0.16 >2.55, <0.27	C0: 290.26 C16: 0.20 C18: 0.15 C18:1: 0.19	C0: 330.88 C16: 0.19 C18: 0.14 C18:1: 0.16	Out of range
2691	Phe	PKU	<68	>109	480.76	694.23	Out of range
3700	Multiple (C6, C8, C10:1, C16)	MCAD	<0.11 <0.1 <0.11 0.41–6.9	>0.14 >0.3>0.21 >7.13, <0.33	C6: 0.56 C8: 1.26 C10:1: 0.49 C16: 0.26	C6: 0.57 C8: 1.67 C10:1: 0.65 C16: 0.32	Out of range
606	Multiple (Leu, Val)	MSUD	<170 <159	>191 >166	Leu: 1511 Val: 363	Leu: 1861 Val: 468	Out of range
831	Gly	NKH	<308.46	>336.58	470.49	520.01	Out of range

## Data Availability

All data are available within this article.

## Supplementary Materials

The following supporting information can be downloaded at: https://www.mdpi.com/article/10.3390/ijns11010004/s1. Table S1: LC-MS/MS parameters for underivatized amino acids and acylcarnitines; Table S2: recovery of amino acids and acylcarnitines in PHUNSA MS/MS kit; Table S3: intra-labortory precision of the PHUNSA kit for selected analytes at two concentration levels; Table S4: multi-site precision of the PHUNSA kit for various analytes across two concentration levels; Table S5: comparison of PHUNSA and MassChrom kits for analyzing control sample I (*n* = 40); Table S6: comparison of PHUNSA and MassChrom kits for analyzing control sample II (*n* = 40); Table S7: kit comparison using 40 real samples for PHUNSA and MassChrom kits (*n* = 40); Table S8: LOD and LLOQ results for the PHUNSA kit (µmol/L); Table S9: linearity and performance data for the PHUNSA kit; Table S10: measured values of blank filter paper samples (µmol/L) and memory effect (µmol/L); Table S11: % deviation from the target for Level I control sample of amino acids for the accelerated stability; Table S12: % deviation from the target for level I control sample of acylcarnitines for the accelerated stability; Table S13: % Deviation from the target for Level II control sample of amino acids for the accelerated stability; Table S14: % Deviation from the target for Level II control sample of acylcarnitines for the accelerated.
