# Supplementary material for: Development, Validation, and Application of the Paya Hamsan Technologies Underivatized Newborn Screening Assay (PHUNSA) for Inborn Metabolic Disorders in Dried Blood Spot Samples from Iranian Infants
Source: Int J Neonatal Screen. 2025 Jan 8;11(1):4. doi: 10.3390/ijns11010004 (PMC11755433; doi:10.3390/ijns11010004)
Supplement: Supplementary file 1 [file IJNS-11-00004-s001.zip › IJNS-3357073-supplementary.pdf]

International Journal of
Neonatal Screening

MDPI

Supplementary Materials

Development, Validation, and Application of the Paya Hamsan Technolgies Underivatized Newborn Screening Assay (PHUNSA) for Inborn Metabolic Disorders in Dried Blood Spot Samples from Iranian Infants

Equipment and Instrument Parameters

LC-Parameter

Pump: Isocratic

Injection volume: 10 µl

Run time: 2 min

Needle rinsing solution for the injector: Rinsing Solution (Wash Time: 5 sec)

Flow gradient: 20 to 500 µl/min

Mobile Phase: Acetonitrile 80/Water 20

MS-Parameter

Ion Source: Electrospray ionisation (ESI), Positive

MS/MS mode: MRM

DP: Declustering Potential

EP: Entrance Potential

CE: Collision Energy

Table S1. LC-MS/MS Parameters for Underivatized Amino Acids and Acylcarnitines.

Amino Acids - Non-Derivatization					
**Analyte**	**Precursor (Q1 Mass (Da))**	**Product (Q3 Mass (Da))**	**Time (msec)**	**DP (Volts)**	**CE (Volts)**
Alanine	90	44	50	7	18
Alanine-d4	94	48	50	7	18
Arginine	175	70	50	7	35
Arginine-d7	182	77	50	7	35
Aspartic acid	134	116	50	7	11
Aspartic acid-d3	137	119	50	7	11
Citrulline	176	113	200	7	22
Citrulline-d2	178	115	200	7	22
Glutamic acid	148	130	50	7	14
Glutamic acid-d5	153	135	50	7	14
Glycine	76	30	50	7	16
Glycine-13C2,15N	79	32	50	7	16
Leucine	132	86	50	7	14
Leucine-d3	135	89	100	7	14
Methionine	150	133	50	7	14
Methionine-d3	153	136	50	7	14
Ornithine	133	70	50	7	25
Ornithine-d6	139	76	50	7	25
Phenylalanine	166	120	50	7	17
Phenylalanine-d5	171	125	50	7	17
Proline	116	70	50	7	22
Proline-d7	123	77	50	7	22

Tyrosine	182	136	50	7	17
Tyrosine-d4	186	140	50	7	17
Valine	118	72	50	7	16
Valine-d8	126	80	50	7	16
**Acylcarnitines and Free Carnitine - Non-Derivatization**					
**Analyte**	**Precursor (Q1 Mass (Da))**	**Product (Q3 Mass (Da))**	**Time (msec)**	**DP (Volts)**	**CE (Volts)**
Carnitine	162	85	50	7	16
Carnitine-d9	171	85	50	7	16
Acetylcarnitine	204	85	50	7	27
Acetylcarnitine-d3	207	85	50	7	27
Propionylcarnitine	218	85	50	7	27
Propionylcarnitine-d3	221	85	50	7	27
Butyrylcarnitine	232	85	50	7	31
Butyrylcarnitine-d3	235	85	50	7	31
Isovalerylcarnitine	246	85	100	7	32
Isovalerylcarnitine-d9	255	85	100	7	32
Glutarylcarnitine	276	85	50	10	32
Glutarylcarnitine-d9	285	85	50	7	32
Hexanoylcarnitine	260	85	50	7	34
Hexanoylcarnitine-d3	263	85	50	7	34
Octanoylcarnitine	288	85	50	7	35
Octanoylcarnitine-d3	291	85	50	7	35
Decanoylcarnitine	316	85	50	7	40
Decanoylcarnitine-d3	319	85	50	7	40
Dodecanoylcarnitine	344	85	50	7	40
Dodecanoylcarnitine-d3	347	85	50	7	40
Tetradecanoylcarnitine	372	85	50	7	46
Tetradecanoylcarnitine-d3	375	85	60	7	46
Hexadecanoylcarnitine	400	85	60	7	48
Hexadecanoylcarnitine-d3	403	85	50	7	48
Octadecanoylcarnitine	428	85	50	7	52
Octadecanoylcarnitine-d3	431	85	50	7	52

Table S2. Recovery of Amino Acids and Acylcarnitines in PHUNSA MS/MS Kit.

Analyte	Control Sample (Level I)				Control Sample (Level II)			
	Target [μmol/L]	Range [μmol/L]	Mean [μmol/L]	Recovery%	Target [μmol/L]	Range [μmol/L]	Mean [μmol/L]	Recovery%
**Amino Acids**								
Alanine	354	163-545	378.87	107.03	736	323-1149	671.15	91.19
Arginine	97	36-158	109.27	112.65	225	115-335	260.23	115.66
Aspartic Acid	110	71-149	102.47	93.16	261	173-350	259.58	99.46
Citrulline	68	48-88	64.76	95.23	300	221-379	262.21	87.40
Glutamic Acid	464	298-630	507.39	109.35	730	502-958	855.76	117.23
Glycine	257	187-327	252.83	98.38	649	456-842	627.52	96.69
Leucine	278	153-403	304.51	109.54	504	335-673	568.36	112.77
Methionine	49	15-83	51.74	105.59	191	76-306	209.00	109.42
Ornithine	230	136-324	237.39	103.21	547	343-751	521.64	95.36
Phenylalanine	121	77-165	132.93	109.86	436	269-603	523.65	120.10
Proline	299	220-378	310.63	103.89	774	475-1074	806.95	104.26
Tyrosine	192	130-254	212.36	110.61	565	381-731	626.41	110.87
Valine	244	153-335	267.61	109.68	424	278-570	508.09	119.83
**Acylcarnitines and Free Carnitine**								
Carnitine (C0)	49.7	27.8-71.7	58.28	117.26	101	63.0-139	121.69	120.49
Acetylcarnitine (C2)	21	14.4-27.6	21.63	102.99	60.10	37.2-83.0	58.90	98.01
Propionylcarnitine (C3)	4.37	2.52-6.22	4.50	102.90	13.00	8.44-17.6	12.49	96.09
Butyrylcarnitine (C4)	0.83	0.38-1.28	0.84	101.53	3.29	1.92-4.66	3.58	108.89
Isovalerylcarnitine (C5)	0.49	0.25-0.73	0.54	110.79	2.17	1.22-3.12	2.23	102.59
Glutarylcarnitine (C5DC)	0.6	0.15-1.05	0.54	90.79	2.60	1.20-4.0	1.16	44.72
Hexanoylcarnitine (C6)	0.45	0.26-0.64	0.41	91.44	2.12	1.35-2.89	1.89	89.11
Octanoylcarnitine (C8)	0.49	0.26-0.72	0.41	84.00	2.17	1.33-3.01	1.82	83.64
Decanoylcarnitine (C10)	0.48	0.29-0.67	0.36	74.97	1.96	1.08-2.84	1.48	75.43
Dodecanoylcarnitine (C12)	0.46	0.2-0.72	0.52	112.25	2.09	1.37-2.61	2.27	108.50
Tetradecanoylcarnitine (C14)	0.48	0.25-0.71	0.44	92.24	2.09	1.24-2.94	1.85	88.53
Hexadecanoylcarnitine (C16)	4.72	2.86-6.58	4.48	94.81	13.20	8.08-18.3	11.72	88.82
Octadecanoylcarnitine (C18)	2.47	1.38-3.56	2.59	104.72	8.28	4.47-12.3	8.70	105.02

Table S3. Intra-labortory precision of the PHUNSA kit for selected analytes at two concentration levels

Analyte	Level I					Level II				
	Repeat-ability CV%	Between Run CV%	Within Day CV%	Between Day CV%	Within Lab CV%	Repeat-ability CV%	Between Run CV%	Within Day CV%	Between Day CV%	Within Lab CV%
Alanine	4.1%	4.7%	6.2%	4.7%	7.8%	1.6%	5.0%	5.2%	2.0%	5.6%
Arginine	2.6%	4.9%	5.6%	3.7%	6.7%	1.5%	4.3%	4.5%	3.7%	5.9%
Aspartic Acid	4.1%	3.6%	5.5%	2.7%	6.1%	3.9%	2.5%	4.6%	5.0%	6.8%
Citrulline	3.7%	5.4%	6.5%	3.5%	7.4%	3.0%	5.5%	6.3%	1.2%	6.4%
Glutamic Acid	5.6%	4.5%	7.1%	3.9%	8.1%	3.2%	2.7%	4.2%	2.7%	5.0%
Glycine	3.0%	4.2%	5.2%	4.0%	6.5%	2.6%	4.8%	5.4%	1.8%	5.7%
Leucine	1.5%	4.8%	5.0%	0.5%	5.1%	0.8%	4.4%	4.5%	2.9%	5.3%
Methionine	3.9%	6.7%	7.8%	2.6%	8.2%	3.7%	4.6%	5.9%	2.8%	6.5%
Ornithine	3.4%	3.7%	5.0%	3.9%	6.4%	1.4%	4.5%	4.7%	5.5%	7.2%
Phenylalanine	3.1%	4.6%	5.6%	2.2%	6.0%	0.7%	4.8%	4.8%	4.2%	6.4%
Proline	2.1%	4.5%	5.0%	1.2%	5.1%	1.5%	5.0%	5.2%	3.5%	6.3%
Tyrosine	3.4%	5.4%	6.4%	3.1%	7.2%	1.4%	4.4%	4.6%	0.0%	4.6%
Valine	3.3%	4.6%	5.7%	2.6%	6.2%	1.2%	4.8%	4.9%	6.7%	8.3%
Carnitine (C0)	5.4%	4.4%	6.9%	2.9%	7.5%	2.2%	5.1%	5.5%	6.3%	8.4%
Acetylcarnitine (C2)	2.3%	5.6%	6.1%	4.8%	7.8%	1.2%	4.5%	4.6%	0.8%	4.7%
Propionylcarnitine (C3)	2.1%	4.7%	5.1%	4.1%	6.5%	1.7%	4.6%	4.9%	0.0%	4.9%
Butyrylcarnitine (C4)	2.8%	4.4%	5.2%	4.6%	7.0%	2.5%	4.3%	5.0%	0.0%	5.0%
Isovalerylcarnitine (C5)	2.4%	5.5%	6.0%	1.6%	6.3%	1.5%	4.8%	5.1%	2.5%	5.6%
Glutarylcarnitine (C5DC)	10.3%	0.0%	10.3%	3.0%	10.7%	8.2%	0.0%	8.2%	2.8%	8.7%
Hexanoylcarnitine (C6)	2.4%	4.6%	5.2%	5.7%	7.7%	1.7%	3.2%	3.6%	2.7%	4.5%
Octanoylcarnitine (C8)	1.9%	4.8%	5.1%	3.7%	6.3%	1.1%	4.5%	4.7%	0.0%	4.7%
Decanoylcarnitine (C10)	1.9%	4.7%	5.0%	4.6%	6.8%	1.0%	4.3%	4.5%	0.0%	4.5%
Dodecanoylcarnitine (C12)	1.8%	5.2%	5.5%	3.3%	6.4%	1.0%	4.3%	4.4%	0.0%	4.4%
Tetradecanoylcarnitine (C14)	1.5%	5.2%	5.4%	3.6%	6.5%	0.9%	4.6%	4.7%	0.0%	4.7%
Hexadecanoylcarnitine (C16)	0.8%	5.0%	5.0%	2.9%	5.8%	0.6%	5.1%	5.1%	0.0%	5.1%
Octadecanoylcarnitine (C18)	0.9%	5.0%	5.1%	2.6%	5.7%	0.6%	5.6%	5.7%	0.4%	5.7%

Table S4. Multi-site precision of the PHUNSA kit for various analytes across two concentration levels

Analyte	Level I					Level II				
	Repeat-ability	Between Day	Within Instru-ment	Between Instu-ment	Total CV%	Repeata-bility	Between Day	Within Instru-ment	Between Instru-ment	Total CV%
Alanine	6.8%	6.0%	9.0%	0.0%	9.0%	6.3%	5.0%	8.1%	6.2%	10.2%
Arginine	7.3%	3.9%	8.3%	1.5%	8.4%	5.6%	2.6%	6.2%	9.5%	11.3%
Citrulline	10.6%	0.0%	10.6%	9.4%	14.2%	6.0%	2.7%	6.5%	8.0%	10.3%
Glutamic Acid	6.3%	0.0%	6.3%	12.7%	14.2%	5.7%	1.3%	5.9%	16.9%	17.9%
Glycine	8.2%	3.3%	8.8%	0.0%	8.8%	7.9%	4.5%	9.1%	0.0%	9.1%
Leucine	7.0%	0.0%	7.0%	11.9%	13.8%	6.1%	4.9%	7.9%	14.3%	16.3%
Methionine	8.8%	2.4%	9.1%	14.7%	17.3%	5.6%	3.8%	6.8%	15.8%	17.2%
Ornithine	5.9%	4.5%	7.5%	21.3%	22.6%	5.9%	1.9%	6.2%	18.7%	19.7%
Phenylalanine	7.2%	2.7%	7.7%	0.0%	7.7%	6.0%	4.0%	7.2%	6.1%	9.5%
Proline	7.3%	1.4%	7.4%	0.0%	7.4%	5.9%	4.8%	7.7%	2.1%	7.9%
Tyrosine	6.2%	2.4%	6.6%	4.4%	8.0%	5.4%	3.3%	6.3%	5.3%	8.2%
Valine	7.6%	0.0%	7.6%	10.1%	12.7%	6.3%	6.1%	8.8%	7.3%	11.5%
Carnitine (C0)	9.6%	1.8%	9.8%	10.4%	14.3%	6.8%	6.3%	9.3%	7.0%	11.6%
Acetylcarnitine (C2)	-	-	-	-	-	7.5%	4.2%	8.6%	7.0%	11.1%
Propionylcarnitine (C3)	7.9%	0.9%	8.0%	6.3%	10.2%	7.0%	4.6%	8.4%	8.3%	11.8%
Butyrylcarnitine (C4)	9.9%	0.0%	9.9%	5.4%	11.3%	7.2%	4.5%	8.5%	7.7%	11.5%
Isovalerylcarnitine (C5)	6.8%	6.4%	9.4%	24.6%	26.4%	5.5%	4.0%	6.8%	19.2%	20.4%
Glutarylcarnitine (C5DC)	7.4%	3.8%	8.3%	11.4%	14.1%	8.9%	3.0%	9.4%	18.4%	20.7%
Hexanoylcarnitine (C6)	11.2%	0.5%	11.2%	20.1%	23.0%	6.8%	5.8%	9.0%	2.1%	9.2%
Octanoylcarnitine (C8)	7.6%	0.0%	7.6%	6.0%	9.7%	7.5%	6.4%	9.9%	2.9%	10.3%
Decanoylcarnitine (C10)	8.6%	0.0%	8.6%	10.9%	13.8%	8.4%	6.7%	10.7%	7.6%	13.1%
Dodecanoylcarnitine (C12)	8.6%	0.0%	8.6%	12.3%	15.0%	8.0%	6.6%	10.4%	10.3%	14.6%
Tetradecanoylcarnitine (C14)	8.5%	0.0%	8.5%	15.2%	17.4%	8.5%	6.8%	10.9%	11.3%	15.6%
Hexadecanoylcarnitine (C16)	7.8%	0.0%	7.8%	3.6%	8.6%	7.8%	5.1%	9.3%	6.3%	11.2%
Octadecanoylcarnitine (C18)	7.8%	2.4%	8.1%	1.0%	8.2%	8.2%	3.4%	8.9%	2.3%	9.2%

*** Level I C2 Carnitine concentration was below the detection limit of the AB SCIEX 3200 instrument and is not reported.**

Table S5. Comparison of PHUNSA and MassChrom kits for analyzing control sample I (n = 40)

Analyte	Target [µmol/L]	Range [µmol/L]	PHUNSA			MassChrom		
			Mean [µmol/L]	%CV	Deviation%	Mean [µmol/L]	%CV	Deviation%
**Amino Acids**								
Alanine	705	324-1086	580.56	7.43	-17.65	609.80	5.94	-13.50
Arginine	66	24-108	59.41	6.44	-9.98	58.69	5.26	-11.07
Aspartic Acid	184	118-250	206.89	5.91	12.44	197.84	6.38	7.52
Citrulline	73	51-94	75.48	7.15	3.40	65.33	7.07	-10.51
Glutamic Acid	856	550-1162	836.83	7.87	-2.24	828.70	6.24	-3.19
Glycine	448	325-570	497.35	6.22	11.02	454.32	5.13	1.41
Leucine	471	259-682	441.77	4.99	-6.21	396.12	5.58	-15.90
Methionine	102	32-173	93.52	8.02	-8.32	88.85	5.75	-12.89
Ornithine	526	310-742	531.04	6.07	0.96	514.96	7.86	-2.10
Phenylalanine	297	189-404	241.50	5.85	-18.69	238.65	5.53	-19.65
Proline	480	353-606	452.15	5.03	-5.80	410.69	6.90	-14.44
Tyrosine	235	159-311	208.18	6.94	-11.41	215.28	5.26	-8.39
Valine	367	230-504	356.70	6.06	-2.81	309.81	7.83	-15.58
**Acylcarnitines and Free Carnitine**								
Carnitine (C0)	55.5	31-80	49.61	7.35	-10.61	42.58	7.57	-23.28
Acetylcarnitine (C2)	21.3	14.6-28	19.82	7.36	-6.95	18.58	5.17	-12.76
Propionylcarnitine (C3)	4.39	2.54-6.24	4.32	6.19	-1.64	3.84	5.84	-12.44
Butyrylcarnitine (C4)	0.93	0.43-1.43	0.98	6.56	5.05	0.82	7.35	-11.63
Isovalerylcarnitine (C5)	0.54	0.27-0.80	0.49	6.15	-8.62	0.47	5.91	-12.89
Glutarylcarnitine (C5DC)	0.53	0.13-0.93	0.66	10.55	24.05	0.59	7.59	11.78
Hexanoylcarnitine (C6)	0.46	0.27-0.65	0.47	7.16	1.89	0.42	5.58	-8.18
Octanoylcarnitine (C8)	0.55	0.29-0.81	0.53	5.99	-4.47	0.48	5.61	-13.13
Decanoylcarnitine (C10)	0.49	0.29-0.68	0.47	6.39	-3.33	0.44	6.53	-9.30
Dodecanoylcarnitine (C12)	0.42	0.18-0.66	0.41	6.18	-1.62	0.39	6.31	-8.18
Tetradecanoylcarnitine (C14)	0.46	0.23-0.68	0.45	6.19	-2.94	0.41	6.13	-11.10
Hexadecanoylcarnitine (C16)	4.34	2.63-6.05	4.25	5.60	-2.07	3.81	6.25	-12.30
Octadecanoylcarnitine (C18)	2.51	1.40-3.62	2.37	5.50	-5.76	2.72	7.08	8.35

Table S6. Comparison of PHUNSA and MassChrom kits for analyzing control sample II (n = 40).

Analyte	Target [µmol/L]	Range [µmol/L]	PHUNSA			MassChrom		
			Mean [µmol/L]	%CV	Deviation%	Mean [µmol/L]	%CV	Deviation%
**Amino Acids**								
Alanine	813	357-1269	655.32	5.43	-19.39	660.86	6.20	-18.71
Arginine	196	100-291	180.98	5.54	-7.66	174.68	5.40	-10.88
Aspartic Acid	416	275-557	422.59	6.34	1.58	379.41	8.51	-8.80
Citrulline	238	175-301	254.52	6.31	6.94	216.20	6.14	-9.16
Glutamic Acid	921	634-1208	918.97	4.79	-0.22	872.96	7.00	-5.22
Glycine	678	476-879	694.00	5.58	2.36	608.86	5.94	-10.20
Leucine	598	397-798	626.71	5.11	4.80	541.91	6.00	-9.38
Methionine	243	97-389	237.42	6.35	-2.30	214.39	5.66	-11.77
Ornithine	793	498-1088	726.36	6.69	-8.40	699.58	7.94	-11.78
Phenylalanine	549	338-760	517.22	5.99	-5.79	489.75	6.35	-10.79
Proline	821	503-1138	823.95	5.98	0.36	729.15	6.68	-11.19
Tyrosine	508	348-668	474.93	4.26	-6.51	469.15	5.37	-7.65
Valine	550	360-740	543.99	7.66	-1.09	453.49	7.86	-17.55
**Acylcarnitines and Free Carnitine**								
Carnitine (C0)	101	62.6-139	97.83	7.78	-3.14	80.41	7.06	-20.39
Acetylcarnitine (C2)	53.6	33.2-74	53.88	4.64	0.53	49.41	5.87	-7.82
Propionylcarnitine (C3)	12.1	7.83-16.3	12.12	4.69	0.19	10.57	6.19	-12.62
Butyrylcarnitine (C4)	4.27	2.49-6.05	4.49	4.75	5.23	3.68	6.18	-13.93
Isovalerylcarnitine (C5)	2.19	1.24-3.14	2.10	5.44	-4.20	1.97	6.67	-9.86
Glutarylcarnitine (C5DC)	2.08	0.95-3.20	1.70	8.01	-18.25	2.03	6.91	-2.23
Hexanoylcarnitine (C6)	1.98	1.26-2.70	2.13	4.27	7.79	1.85	5.76	-6.73
Octanoylcarnitine (C8)	2.11	1.29-2.92	2.18	4.38	3.21	1.89	5.58	-10.21
Decanoylcarnitine (C10)	1.95	1.07-2.83	2.03	4.26	3.89	1.80	5.44	-7.70
Dodecanoylcarnitine (C12)	1.93	1.26-2.59	1.95	4.26	1.23	1.75	5.11	-9.14
Tetradecanoylcarnitine (C14)	1.95	1.16-2.74	1.96	4.37	0.51	1.71	5.88	-12.31
Hexadecanoylcarnitine (C16)	11.3	6.91-15.6	11.66	4.81	3.23	9.95	6.33	-11.90
Octadecanoylcarnitine (C18)	8.49	4.59-12.4	8.04	5.59	-5.30	8.90	6.87	4.86

Table S7. Kit comparison using 40 real samples for PHUNSA and MassChrom kits (n = 40)

Analyte	MassChrom Kit	PHUNSA Kit	%Deviation
**Amino Acids**			
Alanine	141.57	133.93	-5.39
Arginine	22.04	23.66	7.39
Aspartic acid	118.30	107.09	-9.48
Citrulline	14.80	16.38	10.67
Glutamic acid	172.03	188.49	9.57
Glycine	114.66	101.45	-11.52
Leucine	125.16	119.15	-4.80
Methionine	13.10	16.52	26.12
Ornithine	65.48	70.26	7.30
Phenylalanine	30.38	26.21	-13.74
Proline	116.05	103.45	-10.85
Tyrosine	46.64	40.18	-13.84
Valine	66.86	56.36	-15.71
**Acylcarnitines and Free Carnitine**			
Carnitine (C0)	20.86	17.79	-14.71
Acetylcarnitine (C2)	11.35	9.76	-14.01
Propionylcarnitine (C3)	1.14	1.02	-11.03
Butyrylcarnitine (C4)	0.18	0.15	-16.01
Isovalerylcarnitine (C5)	0.12	0.13	5.47
Glutarylcarnitine (C5DC)	0.19	0.24	25.88
Hexanoylcarnitine (C6)	0.06	0.06	-4.89
Octanoylcarnitine (C8)	0.07	0.06	-16.94
Decanoylcarnitine (C10)	0.09	0.07	-19.47
Dodecanoylcarnitine (C12)	0.05	0.04	-9.18
Tetradecanoylcarnitine (C14)	0.08	0.06	-20.33
Hexadecanoylcarnitine (C16)	0.73	0.72	-1.91
Octadecanoylcarnitine (C18)	0.44	0.44	0.66

Other Acylcarnitines			
C4DC&C5OH	0.207	0.149	-28.145
C5:1	0.041	0.030	-26.101
C6DC	0.263	0.301	14.297
C8:1	0.093	0.083	-10.799
C10:1	0.082	0.064	-21.746
C10:2	0.022	0.019	-16.924
C12:1	0.029	0.029	2.154
C14:1	0.047	0.044	-6.421
C14:2	0.020	0.017	-18.240
C14OH	0.006	0.004	-22.685
C16OH	0.007	0.007	-8.527
C16:1	0.037	0.039	4.786
C16:1OH	0.030	0.029	-1.221
C18:1	0.667	0.693	3.805
C18:2	0.235	0.250	6.509
C18:1OH	0.011	0.012	8.872
C18:2OH	0.009	0.010	6.989
C18OH	0.005	0.005	1.191

Table S8. LOD and LLOQ results for the PHUNSA kit (μmol/L)

Analyte	LOD	LLOQ	Analyte	LOD	LLOQ
Amino Acids			Acylcarnitines and Free Carnitine		
Alanine	3.54	10.61	Carnitine (C0)	0.41	1.22
Arginine	1.11	3.33	Acetylcarnitine (C2)	0.29	0.88
Aspartic Acid	2.14	6.42	Propionylcarnitine (C3)	0.03	0.09
Citrulline	2.39	7.17	Butyrylcarnitine (C4)	0.04	0.12
Glutamic Acid	2.97	8.91	Isovalerylcarnitine (C5)	0.01	0.03
Glycine	6.91	20.72	Glutarylcarnitine (C5DC)	0.02	0.06
Methionine	0.77	2.30	Hexanoylcarnitine (C6)	0.01	0.02
Leucine	1.32	3.97	Octanoylcarnitine (C8)	0.00	0.01
Ornithine	4.16	12.48	Decanoylcarnitine (C10)	0.01	0.03
Phenylalanine	1.17	3.51	Dodecanoylcarnitine (C12)	0.02	0.07
Proline	2.69	8.06	Tetradecanoylcarnitine (C14)	0.01	0.02
Tyrosine	1.12	3.35	Hexadecanoylcarnitine (C16)	0.03	0.08
Valine	2.23	6.69	Octadecanoylcarnitine (C18)	0.01	0.03

Table S9. Linearity and performance data for the PHUNSA kit

Analyte	Repeatability (CV%)	Non-linearity (%)	Linear Range (μmol/L)	Analyte	Repeatability (CV%)	Non-linearity (%)	Linear Range (μmol/L)
Amino Acids				Acylcarnitines and Free Carnitine			
Alanine	7.02	-11.75-2.62	50.55-3235.5	Carnitine (C0)	8.36	-9.15-0.77	2.837-337.5
Arginine	8.91	-3.12-12.19	15.61-249.75	Acetylcarnitine (C2)	9.93	-0.46-11.52	0.513-131.4
Aspartic acid	7.74	-3.17-14.85	30.1-481.5	Propionylcarnitine (C3)	6.92	-12.94-0.95	0.179-22.95
Citrulline	8.02	-7.05-3.36	16.8-537.75	Butyrylcarnitine (C4)	7.77	-1.10-7.64	0.273-17.48
Glutamic acid	10.42	-9.65-5.45	64.125-2052	Isovalerylcarnitine (C5)	9.11	-11.32-0.57	0.019-4.973
Glycine	13.92	-1.72-7.91	24.27-3107.25	Glutarylcarnitine (C5DC)	13.21	-4.01-12.56	0.119-0.956
Leucine	7.00	-8.55-1.61	23.24-1487.25	Hexanoylcarnitine (C6)	9.01	-11.95-6.47	0.072-2.295
Methionine	10.77	-5.76-2.46	27.7-886.5	Octanoylcarnitine (C8)	7.80	-6.76-1.17	0.065-4.1625
Ornithine	10.15	-10.66-6.89	31.29-1001.25	Decanoylcarnitine (C10)	8.51	-1.33-8.43	0.04-2.565
Phenylalanine	9.13	-7.5-3.49	52.38-1676.25	Dodecanoylcarnitine (C12)	7.64	-5.78-2.59	0.426-13.635
Proline	6.62	-9.03-4.78	37.76-1208.25	Tetradecanoylcarnitine (C14)	9.79	-0.38-10.71	0.028-7.155
Tyrosine	6.99	-5.95-1.92	39.1-1251	Hexadecanoylcarnitine (C16)	7.13	-13.41-2.20	0.411-26.325
Valine	8.57	-2.76-1.26	38.67-1237.5	Octadecanoylcarnitine (C18)	7.65	-13.76-2.23	0.127-8.145

Table S10. Measured values of blank filter paper samples [μmol/L] and Memory Effect [μmol/L]

Amino Acids			Acylcarnitines and Free Carnitine		
Analyte	Blank Test	Carry Over	Analyte	Blank Test	Carry Over

	Mean	LLOQ	Mean	LLOQ		Mean	LLOQ	Mean	LLOQ
Alanine	8.73	10.61	8.78	10.61	Carnitine (C0)	0.72	1.22	0.63	1.22
Aspartic acid	5.39	6.42	1.65	3.33	Acetylcarnitine (C2)	0.17	0.88	0.15	0.88
Arginine	1.73	3.33	5.25	6.42	Propionylcarnitine (C3)	0.03	0.09	0.03	0.09
Citrulline	1.90	7.17	1.25	7.17	Butyrylcarnitine (C4)	0.02	0.12	0.01	0.12
Glutamic acid	3.56	8.91	3.33	8.91	Isovalerylcarnitine (C5)	0.01	0.03	0.01	0.03
Glycine	12.31	20.72	11.77	20.72	Glutarylcarnitine (C5DC)	0.02	0.06	0.02	0.06
Leucine	2.95	3.97	2.75	3.97	Hexanoylcarnitine (C6)	0.01	0.02	0.01	0.02
Methionine	0.52	2.30	0.48	2.30	Octanoylcarnitine (C8)	0.00	0.01	0.00	0.01
Ornithine	8.91	12.48	9.43	12.48	Decanoylcarnitine (C10)	0.01	0.03	0.01	0.03
Phenylalanine	2.13	3.51	1.92	3.51	Dodecanoylcarnitine (C12)	0.01	0.07	0.01	0.07
Proline	4.81	8.06	4.76	8.06	Tetradecanoylcarnitine (C14)	0.01	0.02	0.01	0.02
Tyrosine	3.48	3.35	3.23	3.35	Hexadecanoylcarnitine (C16)	0.04	0.08	0.04	0.08
Valine	3.38	6.69	3.18	6.69	Octadecanoylcarnitine (C18)	0.01	0.03	0.01	0.03

Stability:

Accelerated stability studies are conducted to increase the rate of chemical degradation or physical change of a material using extreme storage conditions. These studies are used as part of real-time stability studies to express the claimed shelf life.

According to ASTM F 1980-16 standard, the calculation of accelerated study time is based on the Q10 curve and Equations S1 and S2.

$$AAF = Q_{10}{}^{[\frac{T_{AA}-T_{RT}}{10}]} \quad (1)$$

Where, AAF is the Accelerated Aging Factor

Q10 is the reaction (or activation energy)

TAA is the temperature the samples are tested

TRT is the ambient temperature to be assumed

$$AAT = Desired\ RT/AAF \quad (2)$$

Where, AAT: Accelerated Aging Time

RT: Real Time

In the accelerated lifetime testing of the internal standard, samples were stored at temperatures of 2-8°C, 15-30°C, and 45°C for 6 days, and compared to the internal standard stored at -18°C (reference sample). The analysis was conducted using the SHIMADZU LC-MS-MS 8045 and MassChrom control samples. The comparison basis was the reading of the control samples at levels I and II and the measurement of the percentage deviation of readings from the reference sample. The data evaluation was performed using Microsoft Office Excel 2019. The measured concentration results for all analytes, in accordance with the accelerated stability study protocol, are presented in Tables S12-S15.

Table S11. % Deviation from the target for Level I control sample of amino acids for the accelerated stability.

+45 °C												
Day	Ala	Arg	Cit	Glu	Gly	Leu	Met	Orn	Phe	Pro	Tyr	Val
1	-8.76	-8.80	0.89	-1.65	4.60	-7.83	1.19	-9.09	-7.82	-5.62	-12.10	-5.19
6	19.10	18.28	17.13	9.80	11.41	18.01	14.05	11.18	16.34	15.68	12.71	14.50
+22 °C												
Day	Ala	Arg	Cit	Glu	Gly	Leu	Met	Orn	Phe	Pro	Tyr	Val

1	-4.66	-7.35	2.71	-5.53	2.40	-5.55	0.72	-6.22	-7.41	-5.77	-10.20	-4.97
6	3.69	4.17	4.11	1.34	9.17	7.65	9.18	6.22	5.69	7.65	4.83	7.87
+2 - +8 °C												
Day	Ala	Arg	Cit	Glu	Gly	Leu	Met	Orn	Phe	Pro	Tyr	Val
1	-3.38	-7.35	4.88	0.91	7.17	-6.77	4.27	-7.17	-9.09	-6.72	-6.98	-4.91
6	0.02	12.83	-1.92	2.15	3.82	4.94	6.31	2.20	8.34	1.55	5.73	3.74

Table S12. % Deviation from the target for Level I control sample of acylcarnitines for the accelerated stability.

+45 °C													
Day	C0	C2	C3	C4	C5	C5DC	C6	C8	C10	C12	C14	C16	C18
1	-8.41	-12.65	-8.13	-1.62	-4.56	11.40	-7.63	-9.39	-7.88	-5.41	-7.56	-6.39	-7.05
6	18.46	2.06	5.07	8.55	15.32	12.55	7.20	10.46	8.38	8.94	8.51	7.61	10.43
+22 °C													
Day	C0	C2	C3	C4	C5	C5DC	C6	C8	C10	C12	C14	C16	C18
1	-8.02	-8.79	-5.56	-0.63	-6.47	4.13	-3.53	-9.60	-6.73	-6.15	-6.69	-9.29	-9.51
6	4.87	2.91	5.39	6.14	5.48	13.84	2.47	3.47	4.85	3.11	4.76	3.51	3.83
+2 - +8 °C													
Day	C0	C2	C3	C4	C5	C5DC	C6	C8	C10	C12	C14	C16	C18
1	-6.97	-8.59	-6.80	-2.06	-9.64	-9.52	-4.58	-7.01	-5. 66	-5.76	-6.94	-5.54	-3.08
6	15.67	0.75	8.24	6.18	3.65	3.93	1.93	3.50	6.13	-0.07	4.33	2.81	2.56

Table S13. % Deviation from the target for Level II control sample of amino acids for the accelerated stability.

+45 °C												
Day	Ala	Arg	Cit	Glu	Gly	Leu	Met	Orn	Phe	Pro	Tyr	Val
1	0.6	0.26	5.29	1.75	5.59	3.38	2.07	1.04	3.37	2.13	1.68	5.70
6	0.46	-2.86	-1.90	1.87	-2.74	-3.15	-2.37	-2.83	-2.62	0.63	-5.73	-0.98
+22 °C												
Day	Ala	Arg	Cit	Glu	Gly	Leu	Met	Orn	Phe	Pro	Tyr	Val
1	11.73	9.62	10.38	9.25	9.42	11.71	6.51	8.79	10.35	12.95	5.24	11.92
6	0.84	-3.47	2.24	0.37	1.09	-0.33	3.57	-4.03	-1.21	0.92	-0.48	0.34
+2 - +8 °C												
Day	Ala	Arg	Cit	Glu	Gly	Leu	Met	Orn	Phe	Pro	Tyr	Val
1	0.23	-1.51	1.43	1.46	-3.11	-4.08	-11.49	-7.87	-5.04	-1.37	-3.09	-2.38
6	3.74	1.55	-1.87	-3.84	-1.15	-0.89	-0.90	-1.17	0.34	0.85	0.60	-2.05

Table S14. % Deviation from the target for Level II control sample of acylcarnitines for the accelerated stability.

+45 °C													
Day	C0	C2	C3	C4	C5	C5DC	C6	C8	C10	C12	C14	C16	C18
1	2.46	-1.45	1.66	0.59	2.11	-3.49	3.30	0.65	3.51	1.85	-0.18	0.09	-0.82
6	-5.22	-11.81	-9.63	-9.62	5.13	4.53	-5.93	-10.69	-9.27	-10.71	-5.01	-1.96	-4.24

+22 °C												
Day	C0	C2	C3	C4	C5	C5DC	C6	C8	C10	C12	C14	C16	C18
1	8.49	11.19	10.46	9.97	8.71	8.90	14.40	12.20	7.72	8.11	7.33	7.62	7.53
6	-1.11	-2.86	-1.32	-1.75	2.20	9.58	1.29	-0.05	-2.03	-2.29	-0.33	0.15	-2.35
+2 - +8 °C													
Day	C0	C2	C3	C4	C5	C5DC	C6	C8	C10	C12	C14	C16	C18
1	-4.99	-3.67	-3.67	-4.70	-6.16	-9.05	4.08	-5.34	-4.96	-5.29	-7.40	-8.97	-7.66
6	4.22	0.12	-0.72	-3.42	2.49	10.60	-1.25	-0.93	-1.94	-4.20	-0.33	2.85	-1.79

By comparing the results presented in the above tables, no significant decrease (< -15%) in the concentration of any analytes in the internal standard was observed after 6 days of product storage at 45°C. Based on the Q10 curve, the accelerated stability for 6 days at 45°C is equivalent to 480 days of stability at -18°C. Therefore, the product's stability is claimed to be 365 days, equivalent to one year.
